# Temporal Trends in Gestational Diabetes Prevalence, Treatment, and Outcomes at Aarhus University Hospital, Skejby, between 2004 and 2016

**DOI:** 10.1155/2018/5937059

**Published:** 2018-03-15

**Authors:** Per Glud Ovesen, Jens Fuglsang, Mette Bisgaard Andersen, Charlotte Wolff, Olav Bjørn Petersen, H. David McIntyre

**Affiliations:** ^1^Department of Obstetrics and Gynecology, Aarhus University Hospital, Aarhus, Denmark; ^2^Faculty of Medicine, University of Queensland, Brisbane, QLD, Australia; ^3^Danish Diabetes Academy, Odense University Hospital, Odense, Denmark

## Abstract

**Background:**

The prevalence of gestational diabetes (GDM) is increasing worldwide. The most important risk of GDM in pregnancy is excessive fetal growth, increasing the risk of complications during delivery as well as long-term complications like obesity and diabetes in both the mother and the offspring.

**Method:**

All women with GDM who delivered a singleton between 2004 and 2016 were included. The treatment of GDM patients sought to achieve normal blood glucose levels, primarily by diet and exercise. If the glycemic targets were not reached, insulin therapy was initiated. Birth weight and birth weight Z-score was calculated corrected for gender and gestational age at delivery.

**Results:**

The study included 1910 women. The number of GDM women increased significantly each year over the course of the study, as did the proportion requiring insulin therapy. Birth weight and birth weight Z-score fell significantly over the years largely due to a decrease in large for gestational age frequency from 29% to around 19%.

**Conclusion:**

During the last 13 years, the number of women diagnosed with GDM has increased. Furthermore, the proportion of GDM women receiving insulin treatment has increased. The birth weight in diet-treated women has been virtually normal for the last 5 years of the reported period.

## 1. Introduction

The prevalence of gestational diabetes (GDM) has been increasing in many parts of the world, with much of this increase attributed to increasing maternal overweight and obesity during pregnancy and to older age at child bearing [1–3].

The most important risk of GDM in pregnancy is excessive fetal growth. Glucose passes through the placenta to the fetus and consequently the fetus develops hyperinsulinemia. Insulin stimulates fetal growth and the extra glucose is stored as body fat causing macrosomia and increasing the risk of birth trauma, shoulder dystocia, and delivery by caesarean section. Long-term complications include the risk of diabetes in the mother and obesity and diabetes in the offspring. Many reports from the last two decades have outlined such risks, thereby emphasizing the need for attention and intervention to address such complications. In this report, we describe the temporal trends for women with GDM in a Danish university hospital setting.

Differing criteria for GDM diagnosis [4, 5] make global comparisons difficult, but in Aarhus, Denmark, the same criterion for GDM diagnosis (2-hour venous plasma glucose after a 75 g glucose load ≥ 9.0 mmol/L) has been used since 2004 [6]. This retrospective cohort study from the Aarhus University Hospital at Skejby aims to document the temporal trends in GDM prevalence, describe patterns of GDM treatment, and outline the pregnancy outcomes for women with GDM and their babies from 2004 to 2016.

Aarhus University Hospital, Skejby, is the principal secondary and tertiary hospital for the mid region of Jutland. Women diagnosed with GDM requiring insulin therapy are routinely referred from regional hospitals for care at Aarhus University Hospital, Skejby (the area covers some 1.3 mio. persons). Pregnant women from the local uptake area (approximately 325, 000 persons) are referred on diagnosis of GDM. From the regional hospitals, women are referred only when insulin treatment is required or other complications develop, usually around gestational week 30 but with large individual variation. The hospital has between 4500 and 5000 deliveries per year; numbers are increasing slightly over time.

## 2. Materials and Methods

The cohort included all women with GDM who delivered a singleton at Aarhus University Hospital, Skejby between 2004 and 2016.The basis of GDM treatment is glycemic control. At the 1st visit, GDM patients received a glucose monitor and were instructed in its use. Patients were taught to check their glucose level before and 1½ hour after breakfast and dinner. The glycemic targets were <6.0 mmol/L before meals and <8.0 mmol/L 1½ h postprandial. All women received dietary advice from a dietician at the time of diagnosis. Specific nutrition/food recommendations were individualized but based on “the 3 Q's”: Quality, Quantity and freQuency and self-monitoring of blood glucose. Recommended dietary macronutrient composition consisted of 45–60% carbohydrates, 10–20% protein, and a maximum of 40% lipids (Table 1). Quantities were individualized based on clinical parameters including pregestational body mass index, hunger, plasma glucose levels, weight gain, and ketone levels. A minimum of 175 g carbohydrate/day was recommended. Weight loss was not recommended; however, for obese women, modest energy restriction by 30% of estimated energy needs was advised to improve glycemic control without ketonuria and reduce maternal weight gain. Regarding the frequency of meals, carbohydrate was distributed throughout the day in three small-to-moderate-size meals and in between two to four snacks. Because of the increased morning insulin resistance, carbohydrate is often less well tolerated at breakfast than other meals. An evening snack may be needed to prevent accelerated ketosis overnight (Table 2). The intention of advising three small-to-moderate-size meals and two to four snacks was to decrease the risk of high postprandial glucose levels with less glucose excursions and thereby reducing fetal hyperglycemia and thus the risk of macrosomia. All patients were advised to undertake daily moderate physical exercise for 30 minutes.

If the glycemic targets were not reached, insulin therapy was initiated using premixed insulin aspart 30%/insulin NPH 70% (Novomix30) before breakfast and dinner. Insulin doses were adjusted according to blood sugar levels, measurements of HbA1c, and ultrasonographic estimates of fetal growth. When insulin therapy is added to nutrition therapy, a goal is to maintain carbohydrate consistency at meals and snacks to facilitate insulin adjustments.

Patients attended the outpatients' clinic every 2 to 5 weeks. Telephone consultations could replace physical visits, if appropriate. Usually, patients had frequent telephone consultations the first weeks after commencing insulin therapy.

This retrospective cohort study was approved by the Danish Health Authorities (jr. 3-3013-360/1) and the Danish Data Protection Agency (jr. 1-16-02-271-13). Data were extracted from computerized hospital databases, complemented by direct data abstraction from clinical records as required. To facilitate presentation of the data, births were grouped by year of delivery into birth periods as follows: period 1: 2004–2008; period 2: 2009–2012; period 3: 2013–2016.

The majority of descriptive and outcome variables are presented in their original form. Birth weight standard deviation (SD or “Z”) score was calculated as: (Z-score = individual birth weight − mean birth weight/SD birth weight), corrected for gender and gestational age at delivery [7]. Large for gestational age (LGA) and small for gestational age (SGA) were defined as a birth weight Z-score ≥ 1.3 or ≤ − 1.3, respectively. The remainder of the neonates were considered appropriate for gestational age (AGA). Preterm birth was defined as delivery before 37 completed gestational weeks [8].

Data distributions were verified by visual analysis of histograms and Q-Q plots. Sample size for all analyses was sufficient to allow for parametric statistical analysis [9]. Statistical analysis was performed using chi-squared tests for categorical variables (reported as *n* (%)) and analysis of variance for continuous variables (reported as mean (SD)). Statistical significance was accepted at the 5% level on two-tailed testing.

## 3. Results

The study included 1910 women. Numbers of births in each period were as follows: period 1: 2004–2008 (*n* = 497); period 2: 2009–2012 (*n* = 624); period 3: 2013–2016 (*n* = 789). Missing data were more common in period 1 but comprised a maximum of 2% of cases across all variables reported. No data imputation was undertaken. Maternal baseline characteristics are reported in Table 3, and maternal and infant pregnancy outcomes are noted in Table 4.

The number of GDM women treated each year increased significantly over the course of the study, as did the proportion requiring insulin therapy. Interestingly, this cannot be related to increasing maternal overweight/obesity as the mean maternal BMI in GDM women actually fell slightly but significantly over the three periods.

Concerning pregnancy outcomes, the timing of delivery (mean 38.9 weeks) remained constant over time, but the elective caesarean section fell and the simple vaginal delivery rate increased in the periods after 2008. Emergency caesarean section rates remained unchanged at 14% of all deliveries.

**Figure 1:**
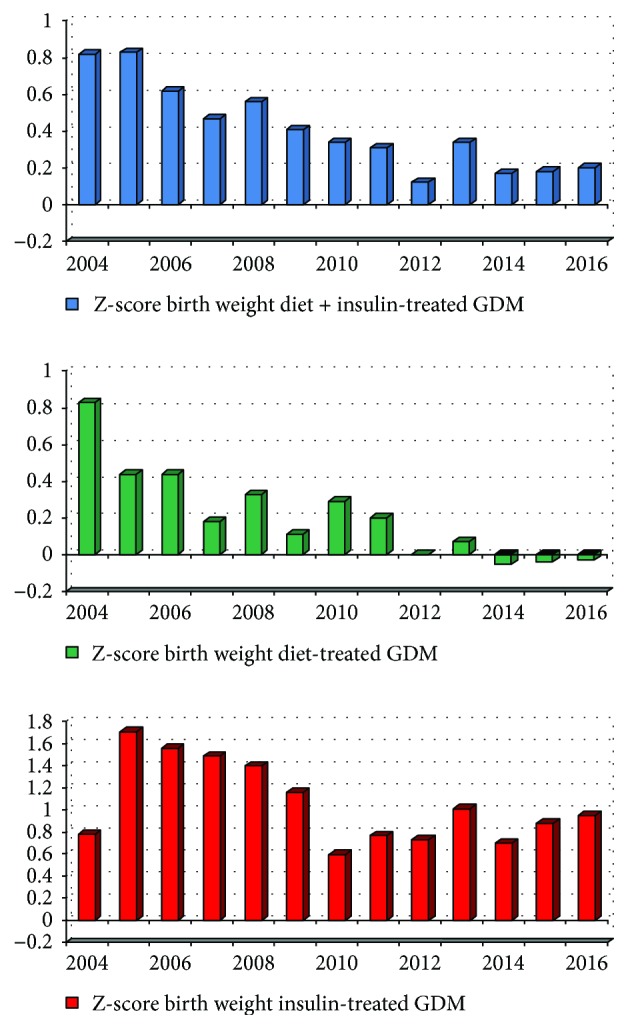
Z-score for birth weight.

Birth weight and birth weight Z-score fell significantly in periods 2 and 3 compared to period 1, with no major changes in head or abdominal circumference. As can be seen in the breakdown of birth weight into SGA/AGA/LGA in Table 3, this was due largely to a decrease in LGA frequency from 29% to around 19%, with a concurrent increase in AGA, but no increase in SGA babies. Similar temporal trends were evident for both diet- and insulin-treated women. Insulin-treated women (mean (SD) BW Z-score: 1.0 (1.5)) had significantly larger babies than those treated with diet alone (BW Z-score 0.2 (1.2); *p* < 0.001) (Figure 1).

To confirm the birth period-related changes noted above, analyses were also performed using year of birth as a grouping variable (rather than birth period). These showed the same temporal trends as noted above (data not shown).

## 4. Discussion

The present data demonstrate that during the last 13 years, the number of women diagnosed with GDM has increased. Furthermore, not only the number but also the proportion of GDM women receiving insulin treatment has increased. Finally, our results demonstrate a decline in the average birth weight in both diet treated and insulin treated GDM women.

### 4.1. GDM Prevalence

The increasing number of women diagnosed with GDM is not unexpected. This phenomenon is known on a large scale not only in industrialized countries but also on a worldwide scale [10, 11]. The reason could be sought in increasing obesity and age among women at child bearing. In our cohort, however, we observed a minor decrease in average BMI among pregnant women with GDM, thus other factors may contribute to our findings. Through the close national obstetrical network [12], knowledge on GDM, its diagnosis, and its consequences for women and their newborns, has been disseminated throughout not only the obstetrical care at the hospitals but even into the nationwide antenatal health care program. Hence, a national awareness concerning GDM exists amongst all caregivers the pregnant women meet in the antenatal program. This vigilance probably does add to the identification and screening of women at risk for GDM. Many women were also referred from neighbouring hospitals for insulin therapy. This number may be expected to increase when overall numbers increase.

### 4.2. Insulin Treatment of GDM

The absolute number and the proportion of women receiving insulin therapy has also increased during the period studied. Increasing absolute numbers would be a natural consequence of the overall increase in women diagnosed with GDM. As the percentages of women receiving insulin also have increased, other explanations must also exist. The average BMI has fallen, primarily due to an increased number of women in the normal BMI category and a decrease in women in the overweight category, rather than to changes in the numbers of women in the extremes of the BMI range (data not shown). Diagnostic criteria for the diagnosis of GDM have not changed in the periods reported.

We hypothesize that increased GDM awareness may have led treating doctors to initiate or adjust insulin therapy not only on the basis of pre and postprandial glucose levels but also due to regular ultrasonographic evaluation of fetal growth/abdominal circumference. It is a well-known clinical experience that some mothers do experience excess fetal growth despite normal or near-normal levels of glucose and HbA1c.

Finally, patients referred from neighbouring hospitals to our tertiary center are those who exhibit the most severe variants of GDM. Centralized treatment of women requiring insulin therapy, introduction of formalized agreements between secondary and tertiary hospitals, and revision of guidelines regarding GDM treatment are all actions that encourage the secondary hospitals to pay more attention to GDM care. Unfortunately, our data do not allow us to identify those patients referred specifically for insulin treatment from neighbouring hospitals. Also, our data do not allow us to discern HbA1c, daily blood sugar levels, or the total daily insulin requirements among pregnant women with GDM. Many pregnant women with GDM maintain a normal HbA1c level for long periods, and the utility of HbA1c as a marker of GDM control is not well established. Future studies should explore if more pregnant women with GDM are put on a low-insulin dose treatment during GDM pregnancy. Finally, one could surmise that as the same team members in our team for pregnant women with diabetes, including the dietician, have been working together for more than 15 years, clinical experience has been accumulated and probably has an impact on daily clinical work.

### 4.3. Birth Weights

Among GDM mothers, the average BW Z-score declined over time. We observed that the number of LGA infants declined with an increase in AGA infants. Thus, the average birth weight tended to normalize without an increase in SGA newborns. We conclude that the effect of interventions has been to normalize birth weights among those GDM women who would otherwise have given birth to the largest babies. This is a preferable outcome.

As can be seen from Figure 1, the birth weight in diet-treated women has been virtually normal for the last 5 years of the reported period. Although the birth weight is decreasing towards normal, some challenges remain in the insulin-treated group. In gestational diabetes, *early* and *sufficient* intervention with insulin is the key to achieve a good neonatal/obstetrical outcome when diet therapy fails to provide adequate glycemic control. Nevertheless, the time frame for treatment is very limited. Many women are reluctant to start insulin therapy, hoping that more intensive dietary management will achieve acceptable glycemic control. Although we do not have data on the gestational week of initiation of insulin therapy, we believe that this postponement of insulin treatment renders the time left to achieve normal birth weight limited. Another obstacle may be slow titration of insulin therapy. This is often commenced cautiously due to concerns about the risk of hypoglycemia, with a consequent delay in achieving optimal glycemic control.

### 4.4. Strength and Weaknesses

For the present data, it is a strength that all data on the DM diagnosis has been reviewed during the pregnancy by one of the two main consultants treating pregnant women with GDM. Birth weights have been captured contemporarily.

Some limitations regarding the diagnosis of GDM have been discussed above as has the pattern in referrals from other hospitals. Furthermore, our source data contain limited information about ethnicity. For example adopted individuals are not readily identified.

### 4.5. External Validity

The present study is a one-center study and thus, in principle, is valid for Aarhus University Hospital, Skejby, only.

As stated above, though, a national societal vigilance on the detection and treatment of GDM is prevailing. National guidelines on the antenatal care and treatment of pregnancies complicated by GDM exist, and the interhospital cooperation between caregivers to GDM pregnant women is high. Thus, the clinical approach is very similar on a nationwide base for the treatment of pregnant women with GDM [13].

## 5. Conclusion

We here present data that show a decline in birth weights towards the normal range in GDM women. Noteworthy is the shift from LGA newborns to AGA newborns without increasing the number of SGA newborns.

At the same time, an increasing number of women are diagnosed with GDM, and an increased proportion of patients with GDM receive insulin therapy.

We speculate that a general societal awareness of GDM has contributed to a better identification of women at risk. Future studies should aim at investigating the patterns among women receiving insulin therapy; especially insulin dosage regimens in relation to birth weights of the neonates deserve to be scrutinized.

## Figures and Tables

**Table 1:** Nutrition therapy for women with gestational diabetes (The 3 Q's).

Nutrition therapy for women with gestational diabetes (The 3 Q's)	
Quality	Protein 10–20%, lipids maximum 25–40%, carbohydrate 45–60%
Quantity	Individualized, but a minimum of 175 g carbohydrate per day
FreQuency	Three small-to-moderate-size meals and two to four snacks

**Table 2:** Example of carbohydrate distribution on a diet of 1800 kcal/day.

	Carbohydrates (g)	% carbohydrates calories
Breakfast	30	15
Morning snack	20	10
Lunch	50	25
Afternoon snack	35	15
Dinner	50	25
Bedtime snack	20	10

**Table 3:** Maternal characteristics.

Variable	Period 1: 2004–2008		Period 2: 2009–2012		Period 3: 2013–2016	
	GDM	All	GDM	All	GDM	All
Total births = 1910	497	21,513	624	17,911	789	18,174
GDM (%)	2.3%		3.5%		4.3%	
Maternal age (years)	32.3	30.0	32.2	30.2	31.7	30.1
Maternal BMI (kg/m^2^)	29.2	23.8	28.1^∗^	23.7	28.0^∗∗^	23.4
Insulin treated *n* (%)	94 (18.9)		127 (20.2)		218 (26.8)^∗^	
Danish born Caucasian (%)	86.5	93.2	85.6	93.6	85.9	94.0

Characteristics of women included in the cohort. Data are presented as mean (SD) or *n* (%).

^∗^ *p* < 0.05 versus period 1; ^∗∗^*p* < 0.01 versus period 1.

**Table 4:** Pregnancy outcomes—mother and infant.

Variable	Period 1: 2004–2008 *n* = 497	Period 2: 2009–2012 *n* = 624	Period 3: 2013–2016 *n* = 789
Gestation at delivery (days)	272 (13)	272 (13)	274 (11)
Delivery mode			
Simple vaginal *n* (%)	306 (61.6)	432 (68.4)	551 (69.8)
Vacuum extraction *n* (%)	0 (0)	2 (0.3)	18 (2.3)^∗^
Elective C-section *n* (%)	109 (21.9)	98 (15.7)^∗^	105 (13.3)^∗^
Emergency C-section *n* (%)	68 (13.7)	85 (13.6)	112 (14.2)
Not recorded *n* (%)	14 (2.8)	9 (1.4)	3 (0.4)
Birth weight (grams)	3605 (582)	3465 (589)^∗∗^	3524 (554)^∗^
Birth weight Z-score	0.64 (1.40)	0.30 (1.35)^∗∗∗^	0.26 (1.27)^∗∗∗^
SGA/AGA/LGA (%)	8/63/29	8/73/19^∗∗∗^	9/73/18^∗∗∗^
Length (cm)	51.9 (2.7)	51.3 (2.8)^∗∗^	51.5 (2.7)
Abdominal circumference (cm)	33.5 (2.2)	33.2 (2.8)	33.3 (2.3)
Head circumference (cm)	35.1 (1.7)	34.9 (1.9)	34.9 (1.7)
Placental weight (g)	718 (174)	673 (187)^∗∗∗^	687(203)^∗∗^
Apgar (5minutes)	9.9 (0.6)	9.9 (0.7)	9.8 (0.8)

Maternal and infant outcomes. Data are reported as mean (SD) for continuous variables and *n* (%) for categorical variables. Statistical comparisons performed using one-way ANVOA followed by unpaired t-tests with Bonferroni correction for continuous variables and Chi squared tests for categorical variables. ^∗^*p* < 0.05 versus period 1; ^∗∗^*p* < 0.01 versus period 1; ^∗∗∗^*p* < 0.001 versus period 1.
